# Strengthening integration of pathways into general practice in Australia: a virtual workshop study with stakeholders

**DOI:** 10.3399/BJGPO.2025.0074

**Published:** 2026-03-11

**Authors:** Faith Yong, Priya Martin, Sneha Kirubakaran, Katharine Ann Wallis, Riitta Partanen, Jordan Fox, Srinivas Kondalsamy Chennakesavan, Matthew McGrail

**Affiliations:** 1 Rural Clinical School, Faculty of Medicine, The University of Queensland, Toowoomba, Queensland, Australia; 2 School of Health and Medical Sciences, University of Southern Queensland, Toowoomba, Queensland, Australia; 3 Rural Clinical School, Faculty of Medicine, The University of Queensland, Rockhampton, Queensland, Australia; 4 General Practice Clinical Unit, Faculty of Medicine, The University of Queensland, Brisbane, Queensland, Australia; 5 Rural Clinical School, Faculty of Medicine, The University of Queensland, Hervey Bay, Queensland, Australia; 6 Allied Health and Human Performance, University of South Australia, Adelaide, South Australia, Australia

**Keywords:** education and standards, qualitative research, workforce development, workforce, general practice

## Abstract

**Background:**

There remains an urgent need for more medical graduates choosing general practice to address expanding GP workforce shortages. Priority interventions remain unclear but strengthened integration of medical training pathways into general practice may help.

**Aim:**

To explore stakeholder views on ways to strengthen integration of pathways into general practice across the medical education and training pipeline.

**Design & setting:**

Participatory research virtual workshop. Participants were purposively sampled to include representation across the Australian medical education and GP training sector.

**Method:**

Using a nominal group technique, participants were prompted to generate suggestions in activities that build on known factors, and to discuss proposed new and modified solutions to the GP shortage. Content analysis and synthesis supported iterative review of the categorisation framework until team consensus was reached.

**Results:**

Seventeen participants (four workshops) made 145 suggestions, which were refined to 67 proposed interventions. There were three overarching categories: (1) improved equity in pay and status for GP trainees and specialists; (2) increased systemic exposure to general practice and generalism; and (3) clearer pathway options to general practice and generalism.

**Conclusion:**

General practice pathways could be strengthened by increased exposure to general practice working conditions and generalism philosophies, along with clearer transitions between different training stages. However, increased equity of resources and the status of general practice within medical specialties were also very important macro factors, beyond education and training alone. Thus, GP pathway interventions require reconfiguration supporting integration into the medical training system to actively value GP healthcare contributions.

## How this fits in

Most countries continue to see too few medical graduates choosing general practice as their specialty. This study sought the views of general practice training stakeholders to answer the question: ‘How can the chief education and training organisations (that is, university, hospital, and GP sector) better integrate training systems to strengthen pathways for uptake of general practice?’. Using participatory research workshops combined with iterative reviewing, we have curated a finalised list of proposed (potential) solutions. This list also highlights many crossovers, thus the need for an integrated approach to support general practice increasing its workforce numbers.

## Introduction

Most countries’ health systems rely on a strong and sufficient primary care (that is, GP) workforce for the wellbeing of the system and their populations.^1^ The GP’s gatekeeper role and oversight of continuous and comprehensive care are key elements to reduce mortality and hospitalisations, along with improved patient management and health system efficiency.^2–4^ However, shortages of GPs have been experienced by most countries.^5^ The recent COVID-19 pandemic was linked to more GPs reducing their work hours or choosing to retire.^6^ In parallel, there is an ongoing trend towards non-GP specialties and subspecialisation of the medical workforce, with insufficient doctors choosing general practice.^7^ Medical teaching and rewarding of specialised knowledge has drifted workforce focus away from the craft of ‘generalism’.^8^

Ideally, medical specialty distribution should align with community healthcare needs. Unlike the UK’s recent experience of insufficient positions to employ trained GPs, Australia generally has insufficient GPs to meet community need.^9^ Surveys confirm only 15%–18% of graduating Australian medical students indicate general practice as their first preference specialty, well short of community need.^10^ Although greater proportions of doctors ultimately chose GP careers (30%–35%), this remains at lower proportions than required.^7^

In this article, pathways into general practice (‘GP pathways’) refers to the experiences, knowledge, and opportunities over the course of their medical training that led medical students and postgraduate doctors to choosing general practice as their medical specialty. Choosing generalist medical careers seems to be greatly influenced by clinical training experiences, as well as their professional and social networks.^11,12^ Most need first-hand data to help them determine if general practice is a good ‘fit’, including whether it is professionally rewarding, suits their practice style, meets their work–life balance goals and desired social position, gains validation from senior clinicians, and enables ruling ‘in’ or ‘out’ of other medical specialty options.^11,13–15^

A noted concern is that direct exposure to GP specialists and their work environments during medical training is often disproportionate to their contribution in the health system.^16^ Globally, early clinical training predominantly occurs in hospital settings,^17^ which may predispose postgraduate doctors towards such career pathways. In countries with prevocational training after medical school, evidence shows this period can strongly influence specialty choices but often occurs siloed from community-based general practice.^18,19^ Importantly, positive GP role modelling (including authentic GP-taught clinical experiences) can favourably shape perceptions and career preferences towards general practice,^16^ while negative role modelling from primary and secondary care sectors can be detrimental.^20^ Unfortunately, stigmatisation against general practice among hospital doctors is common and potentially highly influential, often without balancing voices.^21,22^

There remains an evidence gap of a broader understanding of the role of the key health system components (that is, medical school selection, universities, hospitals, accrediting bodies for attaining GP specialty, and government policy) in shaping career choices and supporting sufficient counts towards general practice. This study aimed to seek the views of general practice stakeholders to identify where and how stronger integration across all sectors of the Australian general practice education and training pipeline is needed. The research question was: *‘*How can the chief education and training organisations (that is, university, hospital, and GP sector) better integrate systems to strengthen pathways for uptake of general practice?’ This study was conducted in Australia, where medical school training occurs over 5–7 years with a university. All graduates then complete prevocational training (commonly 2+ years), predominantly in the hospital setting. GP training is overseen by two organisations, the Royal Australian College of General Practitioners (RACGP) and the Australian College of Rural and Remote Medicine (ACRRM).^23^ This study focuses on the system-level recruitment issues into general practice within Australia.

## Method

This study is phase two of a larger research project. Findings from phase one, involving semi-structured interviews with GPs who had completed general practice training in Australia between 2014 and 2023, are reported separately,^13^ but are used in the analysis phase of this study. This aggregate process brought together expertise and experience from various systems, organisations, and career points. A constructivist paradigm with a systems-level approach was used to conceptualise and understand findings across all Australian medical training stages for GPs, mapping out what factors were necessary for improved uptake of GP careers.^24,25^ The research team took the perspective that the healthcare system and GP training pathways within it constitute a complex adaptive system that is multidimensional, interconnected, and ever-changing.^26^

### Eligibility and recruitment

A broad range of participants were recruited, including clinical and non-clinical staff across most settings for medical training and general practice. This included general practice staff, training programme administrators and managers, medical school educators, GP liaison officers, hospital-based clinical educators, and GP supervisors and educators from both RACGP and ACRRM. GP registrars and medical students were excluded from workshops, in recognition that participants at an early medical career stage were unlikely to have the broad system-level perspectives required for this discussion. Invited participants were selected from key roles at targeted organisations and from the research team’s professional networks. Snowballing from invited participants for further eligible stakeholders was also encouraged. Potential participants were invited via email. Participants were made aware that their responses would be disclosed within their respective workshops and provided written informed consent. Participant willingness and availability and limited study timelines guided recruitment pragmatically.^27^ No compensation was provided for workshop participants.

### Data collection

A 2-hour workshop guide (Supplementary Box S1) for an online focus group with participatory research methodology was developed (by FY). This approach was used to acknowledge and empower stakeholders to contribute their opinions and perspectives.^28,29^ The workshop structure enabled participants to respond, collaborate, and build on ideas, including suggesting and discussing solutions (interventions). Relevant suggestions emerging from phase one were presented to phase two participants as a starting point. Afterwards, using a nominal group technique,^30^ specific prompts and activities were used to ask participants to provide ‘blue-sky’ thinking of solutions supporting an ideal GP pathway, as described in a design-thinking ‘ideation’ phase.^31^ This was counterbalanced through seeking participants to provide additional understanding, objections, or confirmation of the validity of emerging findings, and system-level challenges and facilitators as contextual factors.

Four online workshops were held over 3 weeks in November 2023. These were facilitated via Zoom by two researchers with collective expertise in qualitative research, GP workforce research, and online workshop facilitation (FY and either PM or MM). Workshops were video-recorded and automatically transcribed by Zoom during recording. The workshop co-facilitator made observational notes, while assisting the facilitator to conduct the workshops. The facilitator additionally wrote a debriefing record after each workshop.

### Data analysis

Workshop data were compiled into a spreadsheet. Qualitative content analysis was undertaken by two experienced researchers who were both involved in workshop facilitation (FY and MM).^32^ One researcher (FY) familiarised themselves with the workshop data and compiled a preliminary list of enablers, challenges, and solutions to GP pathways, which underwent participant checking and subsequent modifications. An experienced medical workforce researcher (MM) participated in content analysis, focusing on idea synthesis and alignment with solution categories for clarity and accuracy of intent. Consenting participants provided additional written and verbal perspectives on the direction, accuracy, and wording of the analysis and preliminary findings during design-thinking ‘interpretation’.^33^ The full iterative process of both data collection and analysis (summarised in Table 1) took 3 months.

**Table 1.** Workshop outcomes, analysis stages, and outcomes produced

Data provided to workshop participants	Workshop outcomes	Analysis type	People involved in analysis	Analysis outcomes
Preliminary findings on possible facilitators, barriers, and some proposed solutions to integrating GP education throughout medical training stages	Validation: novel list of facilitators and barriers	1. Summarisation of facilitator or barrier list and participant checking	Researcher (FY), participants	1. Validated summary of facilitators, barriers, and context
		2. Design-thinking ‘interpretation’:^31^ conversion of facilitators and barriers to neutral factors	Researcher (FY)	2. List of neutral factors (including context) affecting GP training integration
		3. Design-thinking ‘prototyping’:^31^ cycles of mind mapping of factors and relationships to each other and participant checking and GP expert review	Researchers (FY and MM), participants with additional consent, GP experts	3. Agreement on factor relationships and complexity, including answering how integrated GP education is within medical training, context for the research question, and how they relate to GP recruitment and retention
		4. Design-thinking ‘interpretation’: rationale for factors in relation to research question and GP expert review and limited participant checking	Researchers (FY and MM), participants with additional consent, GP experts	4. Underlying rationale factors are related to GP training integration agreed on
		5. Design-thinking ‘prototyping’: cycles of visualisation of factors using the rationale as the focus and GP expert review and research team review owing to length of time since initial data collection	Researcher (FY), GP experts, research team	5. Validated visualisation of complex factors around GP training integration in medical training stages
N/A: Nominal group technique used^30^	‘Ideation’: new list of 145 suggestions to create ideal GP training pathway throughout medical training stages, including improving pre-existing supportive structures	6. Qualitative content analysis with several iterative cycles between parties involved over several months^32^ and participant checking and input and GP expert review and research team review	Researchers (FY and MM), participants with additional consent, GP experts, research team	6. Three overall solution categories, 12 summarised solution groups, and reduced list of 67 solutions for readability and action, mapped against validated visualisation from #5

**Figure 1.**
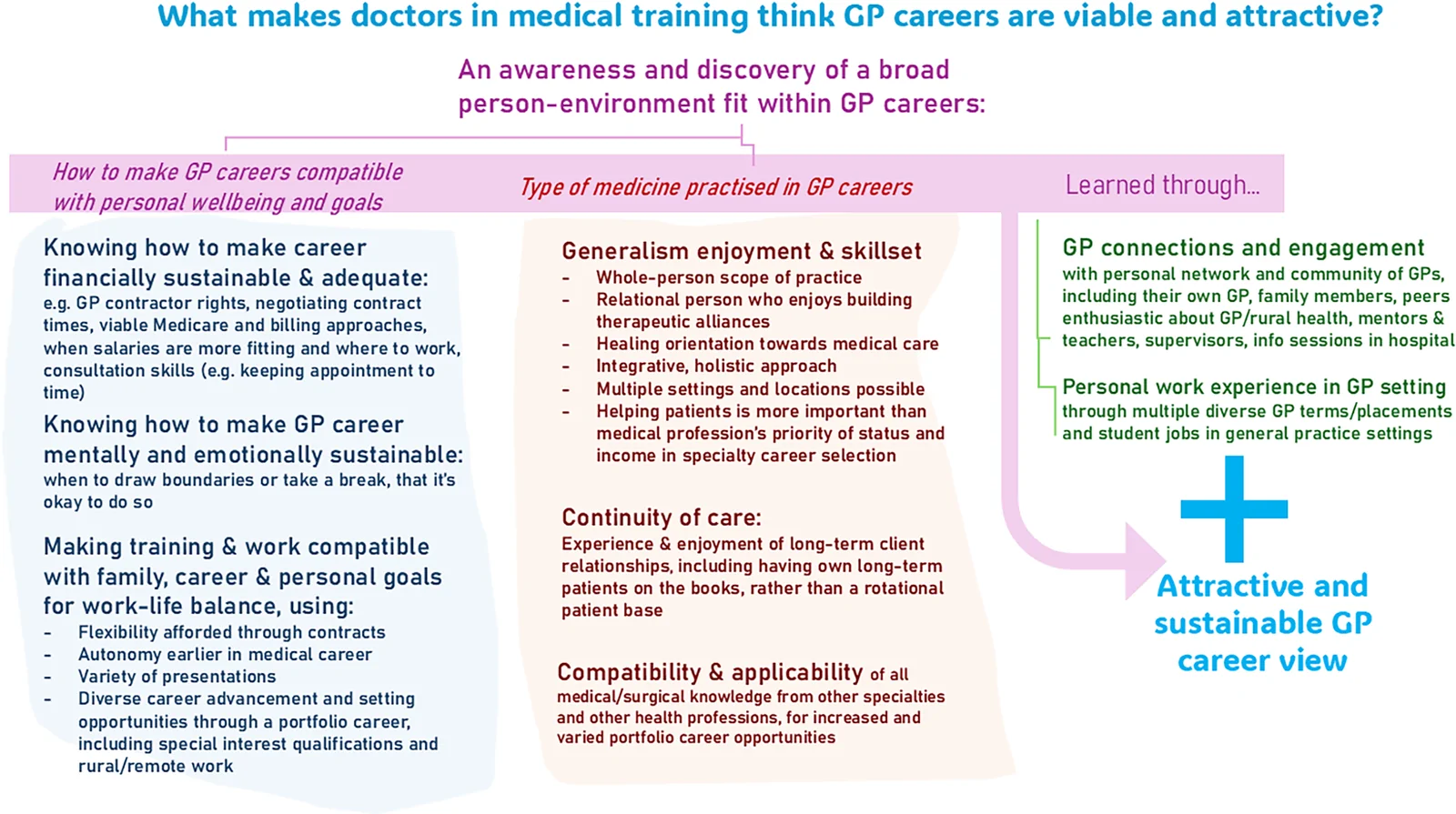
Experiences and knowledge required for conclusions of a sustainable and satisfactory GP career

**Figure 2.**
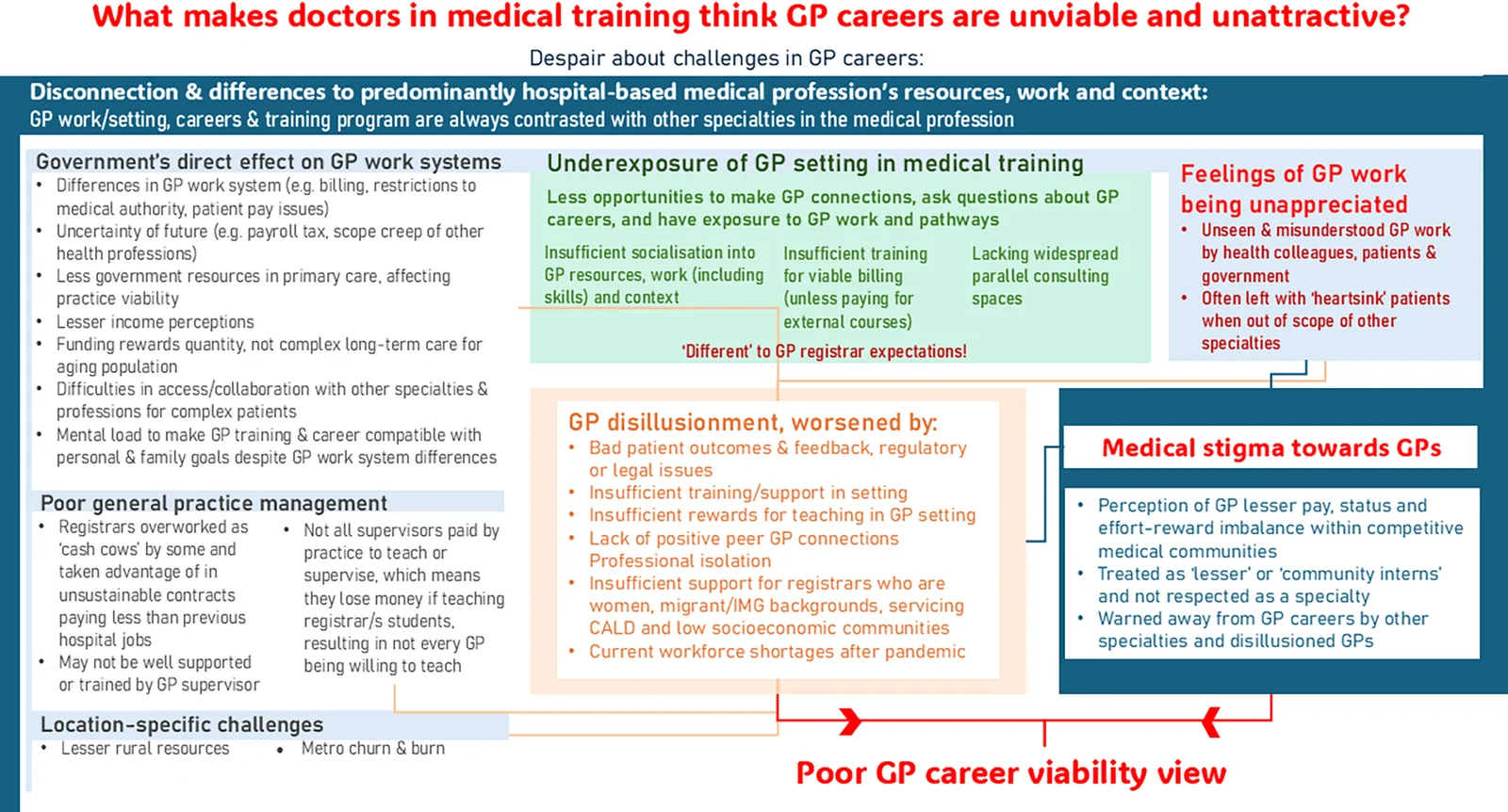
Experiences and knowledge leading to conclusions of a unsustainable and unsatisfactory GP career. CALD = culturally and linguistically diverse. IMG = international medical graduate

A mind-mapping process was undertaken with the collated facilitators and barriers of GP integration into medical training pathways. These were converted to neutral factors experienced by those in GP training and grouped during successive mind-mapping processes. Rationale that provided meaning and associations between the factors was agreed on by consultation with participants, GP experts, and research team members. Complementary to the production of solution lists was the generation of GP career decision viability maps (see Figures 1 and 2). Proposed solutions were mapped against the health system level (macro or meso), educational stage (pre-medicine, medical school, prevocational, registrar training, post-training, all), intervention type (educational, support, incentive, regulatory), and key stakeholders. Proposed solutions underwent further content analysis and similar or overlapping ideas were synthesised. From this, a categorisation framework was created to summarise all GP pathway improvement suggestions. Iterative amalgamation, renaming, and rearranging solutions continued through discussions, grey literature reading, and analysis.

### Trustworthiness and rigour

Several processes were used to ensure trustworthiness and rigour. Optional participant checking of the workshop outcomes (facilitators, barriers, and suggestion lists) was undertaken with phase two participants. Further, preliminary findings were presented to a range of GP education stakeholders during three small group seminars, as well as some phase one participants and GP team members, to obtain input on emerging findings. Reflexive notes were kept by workshop facilitators for inclusion during data analysis (FY and MM or PM). An observational log was kept throughout content analysis for reflexivity. The research team was purposively multidisciplinary to include many perspectives, including researchers familiar with qualitative and participatory methods (FY, PM, SK); researchers with backgrounds in general practice (KW, SK, RP), pharmacy (FY), occupational therapy (PM); and researchers familiar with both medical education and medical workforce distribution challenges (all authors).

## Results

From 28 participants who consented, 17 available participants attended. Workshops lasted for 120 minutes, with two to seven participants per workshop. All medical training sectors were represented with participants including five GP academics, five GP college staff members, six medical educators, two hospital administrators or clinical educators, and one medical school manager (Table 2). Most were medical graduates, resided in Queensland and held multiple roles in different settings.

**Table 2.** Participants of the study workshops

Workshop participant	Sector	Role	Location
01_Wkshp1	University	Medical educator, GP academic and researcher	Metropolitan QLD
02_Wkshp1	University	GP academic and researcher	Metropolitan QLD
03_Wkshp1	RACGP	Medical educator, GP college staff member	Metropolitan NSW and rural NT
04_Wkshp1	ACRRM	GP registrar, GP college staff member	Rural QLD
05_Wkshp2	University	GP clinical educator and supervisor, GP academic and researcher	Metropolitan QLD
06_Wkshp2	RACGP	Medical educator	Metropolitan QLD
07_Wkshp2	ACRRM	Medical educator, GP clinical educator and supervisor	Rural QLD
08_Wkshp3	Hospital	Clinical educator and supervisor in hospital setting	Metropolitan QLD
09_Wkshp3	University	GP academic and researcher	Rural QLD
10_Wkshp3	RACGP	Medical educator	Metropolitan QLD
11_Wkshp3	RACGP	GP college staff member	Metropolitan QLD
12_Wkshp3	RACGP	Medical educator, GP clinical educator and supervisor	Regional centre QLD
13_Wkshp3	RACGP	GP college staff member	Metropolitan QLD
14_Wkshp3	University	GP academic and researcher	Metropolitan QLD
15_Wkshp4	University	Medical school manager	Regional centre QLD
16_Wkshp4	RACGP	GP college staff member	Metropolitan SA
17_NoWkshp	Hospital	Hospital administrator, GP clinical educator and supervisor	Metropolitan QLD

ACRRM = Australian College of Rural and Remote Medicine. NSW = New South Wales. NT = Northern Territory. QLD = Queensland. RACGP = Royal Australian College of General Practitioners. SA = South Australia.

Participants were coded according to workshop participation; the final participant could not attend any of the online workshops but provided feedback to the summary of results sent to all consented participants

Initially, participants summarised the context, reporting on the current status of GP training integration across medical training stages. Overall, they described it as *not* being well integrated in most medical training beyond tokenistic measures, noting that it was not prioritised in clinical situations nor education experiences. In brief, most agreed that GP pathways were largely disconnected from hospital medical learning environments, thus had poor or no visibility to medical students and postgraduate doctors, while general practice was often misunderstood by hospital-based medical seniors. Hope was expressed, with increased availability of quality GP placements to counteract misunderstanding of GP careers and being actively spoken of as having a lesser status; this could also address postgraduate doctors often having little awareness of clinical and administrative processes working in community settings. This process led to collated facilitators and barriers of GP integration into medical training pathways (Supplementary Table S1).

An iterative mind-mapping process was then undertaken with these facilitators and barriers. It became clearer these tended to be associated with both GP uptake *and* retention. Therefore, identified interventions to increase GP workforce numbers needed to address both improved perceptions of GP career sustainability and *actual* GP career sustainability. From this, two differing perspectives were identified by the research team and expanded with participants as underlying the majority of discussions about potential (or needed) solutions. These were as follows:

- viable GP careers (Figure 1): satisfactory and sustainable given specific work knowledge and experience, social connections, and working conditions; and
- unviable GP careers (Figure 2): unsustainable for professional, financial, and personal satisfaction compared with the time, money, or effort expended on them, owing to structural and systemic issues in integrating and prioritising GP-related education.

Figures 1 and 2 summarise the finalised description of experiences and knowledge leading to these two opposing views of the viability of a GP career. These complement the identification of specific solutions in the next section.

### Solutions to better integrate GP pathways across medical training in Australia

Based on the above context, stakeholders attending the workshops made 145 different suggestions to strengthen GP pathway integration within the health system (Supplementary Table S2). Content analysis and refinement of the categorisation framework resulted in the development of three overarching categories, 12 summarised solution groups, and a reduced list of 67 solutions.

These potential ‘solutions’ included past and current interventions, as well as potentially new interventions. They reported facing multiple system-level challenges, which could not easily be solved at the individual practitioner, organisation, or health service levels alone. GP pathways across the health system reportedly needed both top-down and bottom-up change, recognising the need for assistance and support from policymakers and the general public. Proposed solutions were equally likely to be at the macro- or meso-level; most solutions were either supports or educational, rather than incentives or regulatory. Solutions were proportionally spread across all education stages involving a wide range of relevant and responsible stakeholders.

The three solution categories to better integrate GP training throughout medical training stages were as follows: (1) improved equity in pay and status for GP trainees and specialists; (2) increased systemic exposure to general practice and generalism; and (3) clearer pathway options to general practice and generalism.

#### Category 1: Improved equity in pay and status for GP trainees and specialists

Many proposed solutions aimed to address broader professional concerns in general practice primarily driven by perceived inequities within medical specialties, which could hinder the impact of positive changes in GP pathways and career choices. Namely, concerning levels of respect and recognition for GPs needed to be addressed, leading to a strong emphasis on promoting equity in GP pathways and careers.

This ‘improved equity’ category (shorthand ‘EQ’) includes seven summary groups for the proposed solutions. Table 3 has further information on this category and the full list of original suggestions and their mapping to the summary of solutions group(s) is available in Supplementary Table S2.

The seven summary groups are as follows:

**Table 3.** Category 1 (EQ): Improved equity in pay and status for GP trainees and specialists

**EQ 1: Improve GP pay and benefits** Reward GP academics in universities with pay and benefits equivalent to non-GP specialists Provide GP educators and supervisors paid training and support for education in general practice environments (context otherwise lacks support for medical education training) Reward individual GP educators in general practices directly, rather than through general practices Resource general practices to provide medical education, using PIP as lever to upskill organisations Systemise equal pay for GP and non-GP registrars in training programmes (for example, single-employer model and so on) Systemise equal pay for GPs and non-GP specialists Embed GPs in hospitals and other settings to provide GP education, holistic care, and system integrations Re-centre GP work as the 'hub' of Australian health care for system resources, framing hospital care as last resort
**EQ 2: Increase respect for GPs** Ensure respect for GPs is expressed in medical school and medical workplaces Teach respect in medical school and medical workplaces with workplace culture and learning opportunities, for example, GP expert-led continuing professional education Demonstrate respect for GPs systemically (within profession and health system) through equal opportunities available, good-working conditions, and recognition of their work Teach respect for GPs systemically (within profession and health system) by providing learning opportunities about GP integrative and holistic work, enabling greater understanding and appreciation Educate the general public about GP work (particularly integrative, holistic, and system-level mandates) Document what GPs do in research literature (that is, systemic, integrative, holistic, long-term relational work, including hidden care and cognitive work) to strengthen evidence base of the 'value' of GPs to the health system
**EQ 3: Improve GP opportunities: recognition, reward of scope, skills, career ladder** Improved recognition of GP registrar work through increased support Improved recognition of GP role and expertise in multidisciplinary and hospital work Improved recognition for GP educators Recognition of general practice support required Improved recognition of GPs as specialists through pay, see also EQ1 Clear career goalposts; also managing GP scope with skill extensions Managing broad GP scope with multidisciplinary support
**EQ 4: Specific GP training pathway incentives** Subsidised GP exams Special GP trainee positions in prevocational years Collegial work with specialists as GPwSI or GP Improved value proposition compared with hospital work HECS discounts Protected, recognised, and incentivised GP educator positions
**EQ 5: Adjust system structures for less bias against GP training pathways** Resource general practices to be further upskilled as medical education centres for high-quality delivery of GP education for medical students, postgraduate doctors, and registrars, including structural supports enabling educational activities Lessen system bias so it channels less medical students towards non-GP hospital-based rather than GP training Address medical profession bias against GP generalism's contributions to medicine and health system Social accountability measures for medical training stakeholders to assist meeting community needs Reduce system bias from quantity to quality of care in the community, where appropriate Reduce system bias towards community care (compared with hospital care)
**EQ 6: Increase GP leadership and representation in the medical profession, including advocacy for the specialty** GP leadership representation in medical schools aligned with community need GP education and content should be represented in medical training (that is, integrative, holistic, and long-term care) GP role representation in hospitals and other settings to help bridge gaps between primary and tertiary care Education about GP work for policymakers
**EQ 7: Protect GPs and their patients** Resource general practices for medical education, including learner support Remove general practice viability barriers to GP education Reduce GP penalisation for in-depth, complex longitudinal care Make best practice GP medical educators or supervisors in the general practice environment, rewarding quality education Address service gaps, lack of integration between sectors, and overload Clarify the role of primary care and the GP for the general public and policymakers

EQ = ‘increasing equity’ category. GPwSI = general practitioner with special interest(s). HECS = Higher Education Contribution Scheme (university fees). PIP = Practice Incentives Program

1. Improve GP pay and benefits;
2. Increase respect for GPs;
3. Improve GP opportunities, including recognition, reward of scope, skills, and career ladder;
4. Specific incentives for GP training pathways;
5. Adjust system structures for less bias against GP pathways;
6. Increase GP leadership and representation in the medical profession, including advocacy for the specialty; and
7. Protect GPs and their patients.

Participants consistently compared the general practice context with hospital-based non-GP specialties. Such comparisons resulted in conclusions that the GP specialty and setting were disadvantaged in multiple ways, since they were limited by the lesser resources available outside of large hospitals. They felt the contribution, role, and context of being a GP was further misunderstood and maligned by the medical profession, the government, and the general public, which led to ongoing stigmatisation and inequities for GP trainees and fellows:

*‘There is no career pathway for a GP in terms of their income. Let’s get the same* […] *rebate on day one as they do after 20 years of practice! And again, I think that’s a systemic injustice that those of us in leadership around medical education need to highlight to graduates.*’ (Participant 9_Wkshp3, GP academic)

This lack of system integration and understanding of GP work seemed to affect patient care as collateral damage:

*‘… the big problem is the way that health is funded in Australia, which means that GPs get squeezed … which then creates the dissatisfaction and the inability really to offer the kind of care that a lot of GPs would want to, because it just makes no financial sense to do it.*’ (Participant 8_Wkshp3, hospital physician)

Participants noted the need for general practice to be prioritised as a viable sector and career pathway within medical norms, one that also supported the wellbeing of GPs, students, and postgraduate doctors. An analogy was described where GPs were birds who ‘*nested*’ in general practices, with medical students and postgraduate doctors being ‘*eggs*’ within these nests. In the current context, exposing medical students and postgraduate doctors to the realities of the general practice ‘*nest*’ falling to pieces around them risked ‘*eggs being cracked too early*’ and a subsequent lack of succession (Post-workshop discussion, GP team member). The negative undertones of this analogy was supported by concerns raised by other participants:

*‘… something like 78% of GPs would not recommend becoming a GP to doctors coming through —* [we’re] *fighting a very uphill battle. If the people in the seat are telling you not to join, and I think some of the reasons* […] *would be a feeling of disrespect from the government and the community at large.*’ (Participant 10_Wkshp3, GP and RACGP staff)

#### Category 2: Increased systemic exposure to general practice and generalism

Participants advocated for a more systemic exposure to the general practice context throughout medical training, to the philosophy of working as a ‘generalist’ without immediate higher-order support, and to broader structural support for GP medical education. They noted a lack of integration with other medical specialties in hospitals, and suggested exposures should start early and continue at regular touchpoints across the training pipeline.

This ‘increased exposure’ category (shorthand ‘EXP’) includes four summary groups for the proposed solutions. Table 4 has further information on this category and the full list of original suggestions and their mapping to the summary of solutions group(s) is available in Supplementary Table S2.

The four summary groups are as follows:

**Table 4.** Increasing GP exposure and pathway suggestion categories

Category 2 (EXP): Increased systemic exposure to general practice and generalism
**EXP 1: Medical education curriculum and exposure (including GP terms and longitudinal GP terms), solutions assume that GP terms and longitudinal GP terms (including parallel consultations) will continue and increase in scope** GP education content in medical school environments Embed GP-led education into medical school curricula Better promotion, explanation, teaching, and experiences of general practice work GP exposures and connections beyond placements
**EXP 2: Exposure to GPs and GP settings during hospital employment. Assumes prevocational GP-term growth will happen and necessity of voluntary terms plus schemes for rural locations and learning enrichment** Increasing GP education and embeddedness in hospital settings Provide GP roles in hospitals Initiating, maintaining, and enabling GP connections in hospitals Continue voluntary GP opportunities for postgraduate doctors otherwise based in hospitals Provide ongoing support for GP careers in hospitals Provide GP registrar training-specific terms in hospital to ensure learning of specific expertise
**EXP 3: Pre-medical education and broader system-wide interventions** Primary and high school GP career awareness programmes in rural and remote areas with low awareness of medical careers Medical specialty career counselling for a GP endpoint Social accountability measures for medical education and hospital services Facilitating GP-specialist and multidisciplinary teamwork
**EXP 4: Ensuring a safe learning environment in general practice and quality of training** Provide education for GP training preparation Improve GP registrar support structures Improve GP teacher education and support Improve safe learning structures for general practice Through multidisciplinary care models in the community, address GP-overload barriers to GP education that are caused by health system service gaps
**Category 3 (PATH): Clearer pathway options to general practice and generalism**
**PATH: Specific pathways and exposures in medical training to draw individuals to GP careers** Early differentiation in medical education, for example, ADF pathway Rural to GP pathway Prevocational GP pathway, for example, rural generalist pathway GP academic pathway (clinician-educator and/or researcher) Practice ownership pathway

ADF = Australian Defence Force. EXP = ‘increasing exposure’ category. PATH = ‘specific pathways’ category

1. Medical education curriculum and education placements, including longitudinal GP terms and shorter GP placements;
2. Exposure to GPs and GP settings during hospital employment;
3. Pre-medical education and broader system-wide interventions; and
4. Ensuring a safe learning environment in general practice and quality of training.

Senior doctors spoke of GP work being misunderstood within the medical profession, supported by a lack of co-located GP roles within hospital or non-GP specialist roles in GP settings. There was a view that ‘generalism philosophy’, emphasising integrated, whole-person care, which involved understanding individuals and their familial situation over their lifetime, was predominantly practised by GPs, whereas hospital-focused training limited exposure and visibility to this approach. Widespread training in GP contexts, therefore, should strengthen medical training in holistic and individualised approaches to care, which may be comparatively lacking in non-GP specialties with narrower scopes of practice:

*‘I think of “generalism*“ *not just happening in general practice. It can be happening for all good doctors to know how to do generalist thinking, how to see the whole person* […] *especially professions that look after whole families — so palliative care, geriatrics, paediatrics and across into the other disciplines like occupational therapy and social work* […] *that I think we could more intentionally teach early on in medicine as a highly valuable skill* […] *the data of stories and knowing people well.*’ (Participant 9_Wkshp3, GP academic)

#### Category 3: Clearer pathway options to general practice and generalism

A third smaller category was identified that relates to ‘clearer and specific pathways’ into GP specialty (shorthand ‘PATH’). This related to supporting interest through clubs, events, and peer groups and enabling uptake through pathways with fewer barriers. A strong crossover is noted between such pathways and the exposure category (‘EXP’). Table 4 has further information on this category and the full list of original suggestions and their mapping to the summary of solutions group(s) is available in Supplementary Table S2.

Examples of such pathways include alignment of individuals expressing an interest in working in a rural area, military service, generalist field, or small practice or private ownership with choosing a career in general practice.

*‘At the moment, people don't see a clear pathway to general practice.* […] *but if we can create some clearer point* [where] *they can see themselves as a GP in the distant future … see a way to get there, like, you go to XXX Hospital, and then you go to practice A, and then you go to practice B, and then you go to this hospital to gain your advanced skills. Then they don't need to find their own way. And I think that is actually quite attractive.*’ (Participant 3_Wkshp1, GP college rural pathways head and educator)

## Discussion

### Summary

This study used participatory research workshops combined with iterative reviewing to curate a list of general practice training stakeholders' proposals to strengthen integration of pathways into general practice, across the medical education and training pipeline. While many studies highlight the large range of factors that attract students and postgraduate doctors towards general practice,^14,34^ our evidence suggests that systemic inequities, perceptions of GP stigmatisation, and negative commentary regarding general practice within the medical profession may outweigh these factors. A clear set of equity indicators is recommended for addressing GP workforce concerns and to evaluate mitigation strategies.^35^ Other data from those directly involved in GP training confirm similar recommendations, including to: improve GP trainee and educator pay inequities, increase general practice placement opportunities for postgraduate doctors, better integrate general practice and hospital patient care, and address disparaging hospital culture towards general practice.^36^

### Strengths and limitations

The strengths of this study include the use of participatory research design principles that incorporated the lived experiences of recently qualified GP fellows, medical educators, and stakeholders in Australian GP specialty training. However, identified solutions were not all evidence-based nor evaluated for effectiveness; ‘blue-sky’ recommendations were not evaluated against similar interventions internationally. A ‘slow’ approach with incremental change and evaluation is more likely to be helpful owing to the nature of the healthcare system (and primary care) as a complex adaptive system, and we recommend taking cues from intervention design research literature.^37,38^ A series of four workshops is unlikely to create the urgent changes the GP sector desires but is a step in the right direction: the solutions listed require future testing, implementation, and evolution stages to complete the co-design process. One participant group from this process was personnel who had the power and resources to change medical education training on a wider scale. Some workshop participants saw this as a weakness, since moves to implement drastic, bold transformation requires their partnership.

### Comparison with existing literature

Since experiences of a specific specialty can change the trajectory of postgraduate doctors’ career decisions,^39^ it is essential to prioritise systemic exposures to high quality GP experiences for both medical students and postgraduate doctors. Many studies confirm the significant impact of these exposures on choice of general practice careers, although most evidence relates to medical student placements (administered by universities),^40,41^ compared with rotations administered by public hospitals after medical school graduation. Australia’s public and private fragmentations in the health system may have benefits for clinician autonomy and government savings,^42^ but do not translate to accountability and person-centred care. Similarly, the fragmentation of various organisations with differing resources overseeing medical training makes it challenging to align components and encourage more graduates to choose GP careers.

Participants described multiple overlapping disadvantages, including primary care resourcing, professional and physical separation from non-GP specialty colleagues, and other population health challenges, such as patients preferring female GPs for more in-depth and emotionally burdening caseloads. Moreover, higher needs areas — such as rural and remote areas, or those with more socioeconomically deprived communities — experience commensurate 'intersectionality': that is, the presence of one disadvantage stacking on other systemic disadvantages, creating higher strain for the individuals involved.^43,44^ GP occupational strain can be related to perceptions of patient care quality, workload, work–life balance challenges, personal goals attainment, and practice organisation variables.^45–48^ For the GP workforce, this strain poses greater barriers to sustainable and satisfactory provision of quality person-centred care. These factors were compared with other medical specialties and found lacking for GPs in this study, indicating future interventions must take them into consideration.

Multiple recent Australian stakeholder-led reviews conclude the primary care system needs to be better supported.^49–52^ For equitable population coverage, widespread postgraduate doctor exposure to general practice could assist, if subject to appropriate quality guidelines to protect learners, GPs, and patients. The Australian Medical Council (AMC) has introduced changes to the prevocational training framework, which encourage a broader variety of settings including general practice; however, implementation only started in 2024.^53^ The Australian Primary Care Prevocational Program (incorporating the John Flynn Prevocational Doctor Program) has also recently been expanded, supporting rural rollout in general practice; this includes a new second stream supporting widespread metropolitan prevocational non-hospital exposures, beginning in 2026.^54,55^ This also can exclude opportunities for experience in specific populations concentrated in metropolitan areas such as migrant language groups and gender-diversity clinics. Funding remains a key limitation, given Australia’s separation between federal funding for GP training, and state-administered funding for most other specialty and hospital-based training.

### Implications for research and practice

This study addressed evidence gaps regarding the role of key health system components in shaping career choices towards general practice. While aiming to identify improvements and priority solutions, GP stakeholders found it important to describe the state of the sector in greater detail to contextualise their recommendations, indicating that system-driven challenges within the sector are not sufficiently understood or addressed. A united response from the medical profession around the critical nature of GP work, tailoring GP education strategies that are co-created and taught by practising GPs wherever possible would go a long way. It could be that specific and clearer pathways (that is, bundles of interventions that focus on recruiting and retaining specific groups of learners, such as rural students) may also assist. Australia’s recent expansion of the rural generalist pathway builds on this approach, enabling efficient entry points and guaranteed access to the required placement experiences, although outcomes of this are not yet available.^56^

Comparing UK and Australian GP workforces provides some insight into how similar medical training model structures with differing governance can snowball into differing workforce outcomes. Unlike the UK GP funding model, which uses practice patient numbers or block billing, most Australian GPs work privately and ‘bill’ or charge Medicare items (federal funding) according to their clinical discretion, including additional ‘gap’ fees directly to patients. Most Australian GPs function as symbiotic contractors, paying a percentage of their private earnings to their practices for administrative services.^57^ Other differences include that Australia’s Primary Health Networks (PHNs) are not involved in commissioning particular medical services and non-medical practice staff are uncommon outside of nurses and receptionists. Recent efforts by the UK government to increase GP jobs across the country have resulted in increased GP trainees but not increased GP services, leading to a paradoxical crisis of GP unemployment during a time of GP service shortages.^58^ The situation in Australia has been different with its fee-for-service funding model supporting workforce increases, but relies on general practice remaining an attractive option to sufficient postgraduate doctors.

In conclusion, key findings from this study suggest that strengthening the Australian GP pipeline requires improved pay and status equity between general practice and other specialties, increased exposure to general practice and generalism across all stages of medical training, and clearer pathways for those targeting generalist careers. Improved GP education pathways in Australia may require systemic intervention, and an overhaul of all stages and components of medical education and training, enabling general practice to better support provision of high quality experiences for more learners across the training continuum, without compromising patient care. Addressing multifactorial negative cycles of perceived inequities of GP resourcing, remuneration, underappreciation, and stigmatisation will require improved policy leadership in the primary care setting with ongoing collaboration and support from non-GP specialties, universities, state governments, and the federal government.

## Data

The dataset relied on in this article is available from the corresponding author on reasonable request.
